# Morphologic evaluation of meibomian glands in chronic graft-versus-host disease using in vivo laser confocal microscopy

**Published:** 2011-09-29

**Authors:** Yumiko Ban, Yoko Ogawa, Osama M.A. Ibrahim, Yukako Tatematsu, Mizuka Kamoi, Miki Uchino, Saori Yaguchi, Murat Dogru, Kazuo Tsubota

**Affiliations:** 1 Department of Ophthalmology, School of Medicine, Keio University, Tokyo, Japan; 2 Department of Ophthalmology, Hino Municipal Hospital, Tokyo, Japan; 3 Ocular Surface Visual Optics Department, School of Medicine, Keio University, Tokyo, Japan

## Abstract

**Purpose:**

To evaluate the morphological changes of the meibomian glands (MGs) using in vivo laser confocal microscopy (CM) in dry eye (DE) patients with chronic graft-versus-host disease (cGVHD).

**Methods:**

Seventeen eyes from 9 patients with a diagnosis of DE associated with cGVHD (DE/cGVHD group; 6 males, 3 females; median 50.5 years) and 16 eyes of 8 hematopoietic stem cell transplantation (HSCT) recipients without DE (non-DE/non-cGVHD group; 5 males, 3 females; median 47.0 years) were enrolled. CM was used to investigate the MG and MG acinar unit density (MGAUD), MG acinar longest diameter (MGALD), MG acinar shortest diameter (MGASD), and the fibrosis grade. Clinical findings of the lid margin were obtained. Tear dynamics, ocular surface vital staining, meibography, and MG expressibility were also examined. Data were compared between the 2 groups using the unpaired t and Mann–Whitney tests.

**Results:**

The mean MGAUD value was significantly lower in the DE/cGVHD group than in the non-DE/non-cGVHD group (p=0.01, 57.8±38.3 glands/mm^2^, 88.8±26.6 glands/mm^2^, respectively), and the mean MGALD and MGASD were significantly shorter in the DE/cGVHD group than in the non-DE/non-cGVHD group (p=0.0018, 37.3±24.4 μm and 60.4±11.8 μm, p=0.0106, 17.7±11.8 μm and 26.6±6.03 μm, respectively). The mean fibrosis grade was significantly higher in the DE/cGVHD group than the non-DE/non-cGVHD group (p<0.0001, 1.39±0.71 grade, 0.06±0.25 grade, respectively). Clinical findings in the lid margin, tear dynamics, and ocular surface findings were significantly worse in the DE/cGVHD group than in the non-DE/non-cGVHD group.

**Conclusions:**

CM clearly depicted the morphological changes of the MG in the DE/cGVHD group, and revealed the severity of the meibomian gland dysfunction. Patients with severe DE after HSCT showed atrophic MG and excessive fibrosis.

## Introduction

Meibomian glands (MGs) are sebaceous glands embedded within the tarsal plates. Each MG comprises multiple acini connected by a long common central duct running throughout the entire length of the gland. Meibomian glands secrete lipids into the preocular tear film. The meibomian secretions form the outer layer of the tear film to suppress evaporation and function as lubricants for the eyelids during blinking [1]. Meibomian gland dysfunction (MGD) is a chronic, diffuse abnormality of the meibomian glands, commonly characterized by terminal duct obstruction and/or qualitative and quantitative changes in the glandular secretion. This may result in alteration of the tear film, symptoms of eye irritation, clinically apparent inflammation, and ocular surface disease [2].

Chronic graft-versus-host disease (cGVHD) is a major cause of morbidity and mortality in patients undergoing allogeneic hematopoietic stem cell transplantation (HSCT) for hematologic malignancies [3], and MGD is a second most frequent complication of ocular cGVHD. The reported prevalence of MGD in ocular cGVHD is 47.8% [4]. However, to our knowledge, no report on the morphology of MG in cases with cGVHD related dry eye (DE) has been published to date.

Existing methods for assessing MG status and function include slit-lamp examination of the lid margins and ocular surface epithelium, meibometry [5,6] (the assessment of the volume and properties of the meibum), meibography (evaluation of MG morphology, including “drop-outs”) [7-10], and in vivo laser confocal microscopy (CM) [11-13]. The last of these, CM is an emerging new imaging technique that can noninvasively detect structural changes in many ocular surface diseases and anterior segment disorders. In this study, we used CM to investigate the MG of patients with cGVHD-associated DE and compared the results with those from patients who did not develop DE after HSCT.

## Methods

### Subjects and examinations

We had prospectively followed up the HSCT patients at DE outpatient clinic and worked up ocular findings before and after HSCT by communicating with the KEIO BMT program transplant internist since 1996. Confocal microscopy was introduced to our outpatient clinic in 2006. We started this case controlled study since 2010. Between February 2010 and January 2011, we examined 17 patients for this study using CM. Among them, 9 patients had newly developed DE after HSCT. The median period between DE development and HSCT was 22.8 months (range; 8–72 months). Seventeen eyes of 9 patients who had cGVHD related dry eye (DE/cGVHD group; 6 males and 3 females; median age: 50.5 years; range: 38–57 years) and 16 eyes of 8 age- and gender-matched patients who did not develop DE after HSCT (non-DE/non-cGVHD group; 5 males and 3 females; median age: 47.0 years; range: 26–62 years) were analyzed. The demographic and clinical characteristics of the DE/cGVHD group and non-DE/non-cGVHD group are shown in Table 1 and Table 2. There were no statistically significant differences in interval between HSCT and CM analysis between two groups. In addition, we divided the DE/cGVHD group in this study into two groups with or without systemic cGVHD (10 eyes of 5 cases, 7 eyes of 4 cases, respectively) to examine the relationship with systemic cGVHD. The reason why we subdivide the DE/cGVHD group into two groups with or without systemic cGVHD is follows. Conjunctival involvement in GVHD has been reported as a marker for severe systemic GVHD [14]. In our previous study, cGVHD patients with conjunctival fibrosis had systemic complications and a poor prognosis following HSCT [15]. Therefore, we compared between DE/cGVHD group with and without systemic involvement regarding as the meibomian gland acinar unit density (MGAUD), the meibomian gland acinar longest diameter (MGALD) and the meibomian gland acinar shortest diameter (MGASD), and the fibrosis severity.

**Table 1:** Demographic data for patients with chronic GVHD related dry eye (DE/cGVHD group).

**Case**	**Age**	**Gender**	**HSCT source**	**Underlying disease**	**Interval between HSCT and CM analysis (mo)**	**Interval between DE onset and CM analysis (mo)**	**Interval between HSCT and DE onset (mo)**	**DE severity level**	**CC**	**Clinically affected cGVHD organs**
1	57	Male	PBSC	ML	17	1	16	3	-	Eye, Skin
2	47	Male	PBSC	AML	19	6	13	4	+	Eye, Skin, Mouth, Liver
3	50	Male	PBSC	MM	12	1	11	4	+	Eye, Mouth
4	47	Female	BM	MM	43	1	42	4	+	Eye, Mouth
5	38	Female	BM	AML	12	0	12	2	-	Eye
6	57	Male	BM	MDS	14	N.A.	N.A.	3	+	Eye
7	51	Male	BM	CML	120	48	72	2	-	Eye
8	46	Female	PBSC	MM	116	108	8	2	-	Eye
9	55	Male	PBSC	AML	25	17	8	4	+	Eye, Skin

HSCT=hematopoietic stem cell transplantation; CM=confocal microscopy; DE=dry eye; CC=cicatricial change; PBSC=peripheral blood stem cell; BM=bone marrow; ML=malignant lymphoma; AML=acute myeloid leukemia; MM=multiple myeloma; MDS=myelodysplastic syndrome; CML=chronic myeloid leukemia; N.A.=not applicable.

**Table 2:** Demographic data for HSCT recipients without DE (non-DE/non-cGVHD group).

**Case**	**Age**	**Gender**	**HSCT source**	**Underlying disease**	**Interval between HSCT and CM analysis (mo)**
1	55	Male	BM	MM	20
2	28	Male	BM	ALL	168
3	52	Male	PBSC	CML	24
4	62	Male	BM	AML	72
5	26	Male	BM	MDS	26
6	61	Female	BM	AML	39
7	44	Female	BM	CML	199
8	48	Female	BM	AML	177

HSCT=hematopoietic stem cell transplantation; CM=confocal microscopy; DE=dry eye; PBSC=peripheral blood stem cell; BM=bone marrow; MM=multiple myeloma; ALL=acute lymphocytic leukemia; CML=chronic myeloid leukemia; AML=acute myeloid leukemia; MDS=myelodysplastic syndrome.

The definition of systemic GVHD is based on accompanied with or without clinically affected organs other than the eyes as mentioned in the Table 1 and is according to a previous report [16]. We excluded one eye from a patient (Case 5; Table 1) in the DE/cGVHD group because of phthisis from all of the analysis of this study. Topical eye drops, including artificial tears, vitamin A, and autologous serum drops, were instilled five times a day, immediately after the diagnosis of DE following HSCT.

We used the global diagnostic criteria for DE, which is based on the recommendation of the 2007 International Dry Eye Workshop Report [17]. Patients who had a history of surgical lacrimal punctal occlusion, allergies, contact lens use, glaucoma eye drop use, or other ocular surgery, including refractive surgery or radiation to the eyes, were excluded, as were patients with infectious blepharitis, blink disorders, disorders of the lid aperture or lid/globe congruity, or other ocular surface disorders. In addition, patients with trachoma and ocular cicatricial pemphigoid were excluded. The research procedures in this study followed the tenets of the Declaration of Helsinki Principles, and informed consent was obtained from all subjects. IRB/Ethics Committee approval at Keio University was obtained for this study.

### Ocular surface vital staining

The fluorescein and Rose-Bengal staining scores for the ocular surface were obtained using the double vital staining method [18]. Both stains were scored on a scale of 0–9 [18,19]. The van Bijsterveld scoring system was used for the Rose-Bengal staining. Briefly, the ocular surface was divided into three zones: nasal conjunctival, corneal, and temporal conjunctival areas. A score of 0–3 points was used for each zone, with a minimum possible score of 0 and a maximum of 9 points. Scarce punctuate staining was given 1 point, denser staining that did not cover the entire zone was given 2 points, and Rose-Bengal staining over the entire zone was given 3 points. For the fluorescein staining, the cornea was divided into three equal zones (upper, middle, and lower). A score of 0 to 3 points was used for each zone, as with the Rose-Bengal stain. The presence of scarce staining in a zone was scored as 1 point; frequent puncta not covering the entire zone as 2 points, and punctuate staining covering the entire zone as 3 points.

### Meibomian gland secretions

The expression of meibomian secretion (meibum) was graded as described by Shimazaki et al. [10]. Briefly, to assess obstruction of the meibomian gland orifices, digital pressure was applied on the upper tarsus, and the expression of meibomian secretion (meibum) was scored semiquantitatively as follows: grade 0, clear meibum, easily expressed; grade 1, cloudy meibum, expressed with mild pressure; grade 2, cloudy meibum, expressed with more than moderate pressure; and grade 3, no meibum expressed even under hard pressure. Two independent investigators (Y.B. or Y.O.) pressed gently on the upper eyelids to express the meibomian lipids.

### Meibomian gland dropout grades (meibography)

The meibography apparatus was composed of a slit lamp (RO 5000; Rodenstock, Munich, Germany) and an infrared charge-coupled device video camera (XC-EI50; Sony, Tokyo, Japan) [7-9].The MG structure was observed on the under side of the upper and lower eyelids, by everting the lids manually. The degree of MG drop out was scored as reported previously (Shimazaki grading): 0, no gland drop out; 1, drop out with loss of less than half of the glandular structures; and 2, drop out with loss of more than half of the glandular structures [20]. We obtained the values as the average of the upper and lower grades.

### Lid margin abnormalities

The presence or absence of four parameters in lid margin abnormalities (i.e., irregular lid margin; absence=0 or presence=1, vascular engorgement; absence=0 or presence=1, plugged MG orifices; absence=0 or presence=1 and anterior or posterior replacement of the mucocutaneous junction, absence=0 or presence=1) were scored from 0 through 4, according to the number of these abnormalities using slit lamp examination [6,7,21-23]. Therefore, all four parameters for lid margin abnormality were present, the maximum score was given as 4.

### Tear function test

Tear film break up time (TBUT) was measured three times, and the median value was calculated [18]. The Schirmer’s test was performed using standard strips (Showa Yakuhin Kako Co. Ltd., Tokyo, Japan) placed in the lower conjunctival sac for 5 min without anesthesia.

### In vivo laser confocal microscopy

In vivo laser CM was performed on all subjects, using a newly developed confocal microscope, the Heidelberg Retina Tomograph II-Rostock Cornea Module (Heidelberg Engineering GmbH, Dossenheim, Germany), as described previously [11-13]. Briefly, after the lower eyelid was everted, the center of the Tomo-Cap was placed onto the palpebral conjunctiva, and the MGs were scanned while moving the applanating lens. To evaluate the morphologic changes in the MG, we measured the acinar unit density, the longest and the shortest acinar unit diameters, as described previously [11-13]. In addition, we devised one new parameter, the fibrosis severity. The degree of fibrosis severity was scored as follows: grade 0, no fibrosis; grade 1, fibrosis in less than half of the lower eyelid; grade 2, fibrosis in more than half of the lower eyelid. Clearly visible acinar units in a 400×400-μm frame were all counted, and the acinar density was described as the number of units per square millimeter. We calculated the longest and shortest diameters in μm using ImageJ software (Java software program developed by the National Institutes of Health, Bethesda, MD). Three randomized nonoverlapping high-quality digital images of the lower eyelid were used for the CM-based assessments. We calculated the mean MGAUD, MGALD, MGASD, and the fibrosis grade from three randomized nonoverlapping images.

### Diagnosis of dry eye (DE)

The diagnosis and classification of DE disease based on its severity was performed as recommended by the 2007 International Dry Eye Workshop Report. Dry eye is a multifactorial disease of the tears and ocular surface that results in symptoms of discomfort, visual disturbance, and tear film instability with potential damage to the ocular surface. It is accompanied by increased osmolarity of the tear film and inflammation of the ocular surface [17].

### Diagnosis of cGVHD

All the patients in our study fulfilled the revised consensus criteria for cGVHD [24]. Briefly, a diagnosis of cGVHD requires the following: (1) a distinction from acute GVHD, (2) the presence of at least one distinctive manifestation (e.g., keratoconjunctivitis sicca) confirmed by pertinent biopsy or other relevant tests (e.g., Schirmer’s test) in the same or other organs, and (3) the exclusion of other possible diagnoses.

### Conjunctival fibrosis

We diagnosed conjunctival fibrosis in patients who had subconjunctival fibrosis, fornix shortening, symblepharon, and/or ankyloblepharon [14,25]. We evaluated these findings by using slit-lamp microscopy during a routine examination.

### Statistical analyses

The unpaired *t*-test for TBUT, Schirmer test value, MGAUD, MGALD, and MGASD and nonparametric Mann–Whitney test for fluoresecein score, Rose-Bengal score, MG expressibility, meibography, lid margin abnormality and fibrosis grade were used to compare between two groups (the DE/cGVHD group versus the non-DE/non-cGVHD group; Table 3 and Table 4). We also compared between the DE/cGVHD group with and without systemic cGVHD using this test (Table 5). In addition, we compared between the DE/cGVHD group without systemic cGVHD and the non-DE/non-cGVHD group (Table 6). A p value of 0.05 was considered statistically significant. GraphPad Instat (GraphPad Software, San Diego, CA) was used for the statistical analyses.

**Table 3:** Comparison of ocular surface/tear dynamics between the DE/cGVHD group and non-DE/non-cGVHD group.

**Examinations**	**DE/cGVHD group**	**Non-DE/non-cGVHD group**	**p-value**
F (points)	4.88±3.06	0.38±0.62	<0.0001‡
RB (points)	5.06±2.41	0.13±0.34	<0.0001‡
MG expressibility (points)	2.47±0.92	0.75±0.93	<0.0001‡
Meibography (grade)	1.41±0.80	0.50±0.52	0.0014‡
Lid margin (grade)	2.94±1.35	0.38±0.72	<0.0001‡
TBUT (seconds)	2.35±1.90	6.94±3.04	<0.0001*
Schirmer (mm)	1.64±1.21	5.80±2.30	<0.0001*

DE=dry eye; cGVHD=chronic graft-versus-host disease; F=fluorescein score; RB=Rose-Bengal score; MG=meibomian gland; TBUT=tear film break up time; *=Unpaired *t*-test, ‡=Mann–Whitney test.

**Table 4:** Comparison of meibomian glands between the DE/cGVHD group and non-DE/non-cGVHD group using in vivo laser confocal microscopy.

**Examinations**	**DE/cGVHD group**	**Non-DE/non-cGVHD group**	**p-value**
MGAUD (glands/mm^2^)	57.8±38.3	88.8±26.6	0.01*
MGALD (μm)	37.3±24.4	60.4±11.8	0.0018*
MGASD (μm)	17.7±11.8	26.6±6.03	0.0106*
Fibrosis (grade)	1.39±0.71	0.06±0.25	<0.0001‡

DE=dry eye; cGVHD=chronic graft-versus-host disease; MGAUD=meibomian gland acinar unit density; MGALD=meibomian gland acinar longest diameter; MGASD=meibomian gland acinar shortest diameter; *=Unpaired *t*-test; ‡=Mann–Whitney test.

**Table 5:** Comparison of meibomian glands between the DE/cGVHD group with systemic cGVHD and the DE/cGVHD group without systemic cGVHD using in vivo confocal microscopy.

**Examinations**	**DE/cGVHD group with systemic cGVHD (10 eyes, 5 cases)**	**DE/cGVHD group without systemic cGVHD (7 eyes, 4 cases)**	**p-value**
MGAUD (glands/mm^2^)	38.4±30.0	85.5±32.3	0.0097*
MGALD (μm)	19.3±7.31	63.0±14.2	< 0.0001*
MGASD (μm)	10.7±4.33	27.6±12.0	0.0009*
Fibrosis (grade)	1.87±0.23	0.71±0.59	0.0007‡

DE=dry eye; cGVHD=chronic graft-versus-host disease; MGAUD=meibomian gland acinar unit density; MGALD=meibomian gland acinar longest diameter; MGASD=meibomian gland acinar shortest diameter; *=Unpaired *t*-test; ‡=Mann–Whitney test.

**Table 6:** Comparison of meibomian glands assessed by in vivo laser confocal microscopy, MG expressibility, meibography and lid margin abnormality between the DE/cGVHD group without systemic cGVHD and non-DE/non-cGVHD group.

**Examinations**	**DE/cGVHD group without systemic cGVHD (7 eyes, 4 cases)**	**non-DE/non-cGVHD group (16 eyes, 8 cases)**	**p-value**
MGAUD (glands/mm^2^)	85.3±32.3	88.8±26.6	0.8021
MGALD (μm)	63.0±14.2	60.4±11.8	0.65
MGASD (μm)	27.6±12.0	26.6±6.03	0.7888
Fibrosis (grade)	0.71±0.59	0.06±0.25	0.0016‡
MG expressibility (points)	2.20±1.10	0.75±0.93	0.0189‡
Meibography (grade)	1.14±0.69	0.50±0.52	0.0399‡
Lid margin (grade)	2.0±1.41	0.38±0.72	0.0019‡

DE=dry eye; cGVHD=chronic graft-versus-host disease; MGAUD=meibomian gland acinar unit density; MGALD=meibomian gland acinar longest diameter; MGASD=meibomian gland acinar shortest diameter; MG=meibomian gland; ‡=Mann–Whitney test.

## Results

### Data on tear function and ocular surface evaluation

The mean value of tear function test including TBUT and Schirmer test and ocular surface staining scores including Rose-Bengal and fluoresecein were significantly worse in the DE/cGVHD group than in the non-DE/non-cGVHD group (p<0.0001; Table 3).

### Meibomian gland secretions

The mean MG expressibility score was significantly higher (grade 2.47±0.92 grade) in the DE/cGVHD group than in the non-DE/non-cGVHD group (grade 0.75±0.93 grade; p<0.0001; Table 3).

### Meibography

**Figure 1:**
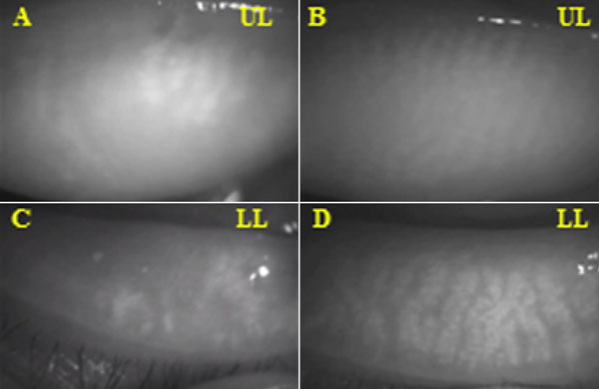
Meibomian gland images observed by noncontact infrared meibography. **A**, **C**: DE/cGVHD group, 57 year-old male (Case 1; Table1). Note the numerous meibomian gland dropouts. **B**, **D**: Non-DE/Non-cGVHD group, 28-year-old male (Case 2; Table2) No loss of meibomian glands was observed in the patient who did not develop DE after HSCT. UL=Upper, Left, LL=Lower, Left.

The numerous meibomian gland dropouts were observed by using noncontact infrared meibography from a 57-year-old male (Case 1; Table 1) in the DE/cGVHD group (Figure 1A,C). In contrast, there was no loss of MG in a 28-year old male (Case 2; Table 2) who did not develop DE after HSCT in the non-DE/non-cGVHD group (Figure 1B,D). The mean meibomian gland dropout grade was significantly higher (1.41±0.80 grade) in the DE/cGVHD group than in the non-DE/non-cGVHD group (0.50±0.52 grade; p=0.0014; Table 3).

### Lid margin abnormalities

**Figure 2:**
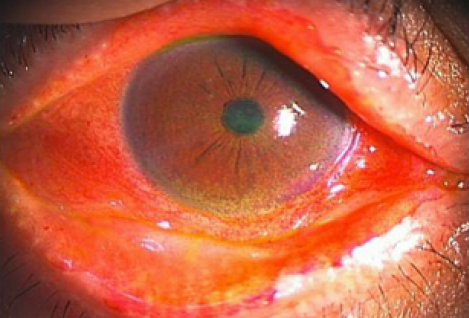
Anterior slit lamp photograph; DE/cGVHD group, 50-year-old male (Case 3; Table 1). The anterior slit lamp photograph showing irregular lid margin, vascular engorgement, plugging of MG orifices, and posterior replacement of the mucocutaneous junction.

Slit lamp examination revealed irregular lid margin, vascular engorgement, plugging of MG orifices and posterior replacement of the mucocutaneous junction from a representative 50-year-old male (Case 3; Table 1) in the DE/cGVHD group (Figure 2).

The mean lid margin score was significantly higher (2.94±1.35 grade) in the DE/cGVHD group than in the non-DE/non-cGVHD group (0.38±0.72 grade; p<0.0001; Table 3).

### In vivo laser confocal microscopy

**Figure 3:**
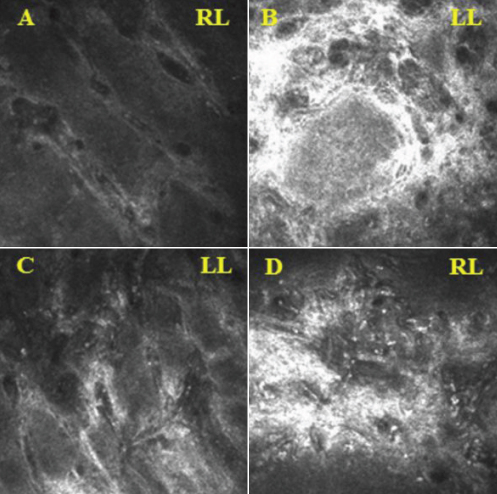
Meibomian gland of DE/cGVHD patients images observed by in vivo laser confocal microscopy. **A**, **C**: DE/cGVHD group, 47-year-old female (Case 4; Table1). **B**, **D**: DE/cGVHD group, 47-year-old male (Case 2; Table 1). The images observed at the onset of DE related to cGVHD. Note the excessive fibrosis around the atrophic glands and the moderate infiltration of inflammatory cells in the dry eye patients with cGVHD. RL=Right, Lower, LL=Left, Lower.

**Figure 4:**
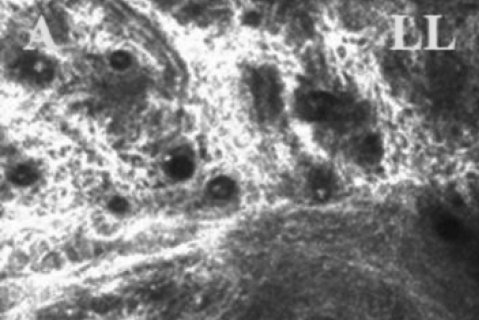
Meibomian gland of DE/cGVHD patient images observed by in vivo laser confocal microscopy. DE/cGVHD group, 55-year-old male (Case 9; Table 1). The images observed after 17 months on the onset of DE related to cGVHD. Note the excessive fibrosis around the atrophic glands and the mild infiltration of inflammatory cells in the dry eye patients with cGVHD. LL=Lower, Left.

**Figure 5:**
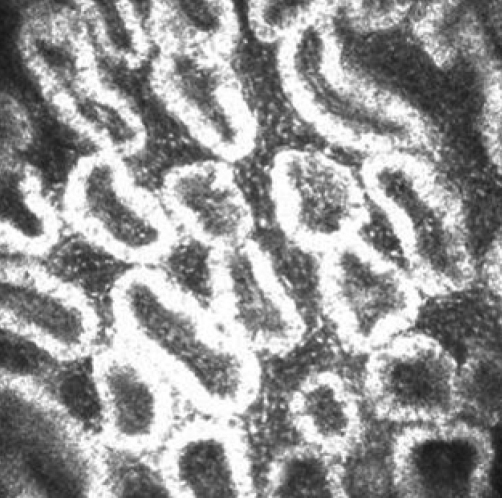
Meibomian gland of non-DE/non-cGVHD recipient images observed by in vivo laser confocal microscopy. Non-DE/Non-cGVHD group, 61-year-old female (Case 6; Table 2). Note the presence of numerous and compact acinar units in patients without dry eye after HSCT.

CM clearly depicted the glandular acinar units in the non-DE/non-cGVHD group. The CM images also revealed morphologic alterations in the DE/cGVHD group, including atrophic MG and extensive fibrosis. The confocal microscopic images observed at the onset of DE related to cGVHD from a representative 47-year-old male (Case 2; Table 1) in the DE/cGVHD group showed the excessive fibrosis around the atrophic glands and the moderate infiltration of inflammatory cells (Figure 3B,D). Seventeen months after the onset of DE related to cGVHD showed the excessive fibrosis around the atrophic glands and the mild infiltration of inflammatory cells from a representative 55-year-old male patient (Case 9; Table 1) in the DE/cGVHD group (Figure 4). In contrast, the confocal microscopic images from a representative 61-year-old female (Case 6; Table 2) in non-DE/non-cGVHD group showed the presence of numerous and compact acinar units (Figure 5).

The mean MGAUD was 57.8±38.3 gland/mm^2^ in the DE/cGVHD group, which was significantly lower than in the non-DE/non-cGVHD group (88.8±26.6 gland/mm^2^, p=0.01). The MGALD was significantly shorter in the DE/cGVHD group than in the non-DE/non-cGVHD group (37.3±24.4 μm versus 60.4±11.8 μm, p<0.0018). In addition, the MGASD was also significantly shorter in the DE-cGVHD group than in the non-DE/non-cGVHD group (17.7±11.8 μm versus 26.6±6.03 μm, p=0.0106). The fibrosis grade was significantly higher (1.39±0.71 grade) in the DE/cGVHD group than in the non-DE/non-cGVHD group (0.06±0.25 grade, p<0.0001, Table 4).

When the DE/cGVHD group was subdivided into patients with systemic cGVHD and patients with only ocular cGVHD, the mean MGAUD value was significantly lower in the patients with systemic cGVHD (38.4±30.0 gland/mm^2^) than in those without it (85.5±32.3 gland/mm^2^, p=0.0097). The MGALD was significantly shorter in the patients with systemic cGVHD than in those without systemic GVHD (19.3±7.31 μm versus 63.0±14.2μm, p<0.0001). In addition, the MGASD was also significantly shorter in the patients with systemic cGVHD than in those without systemic GVHD (10.7±4.33 μm versus 27.6±12.0 μm, p=0.0009; Table 5). The fibrosis grade was significantly higher (1.87±0.23 grade) in the DE/cGVHD group with systemic cGVHD than in those with non-systemic cGVHD (0.71±0.59 grade, p=0.0007; Table 5).

The value of the MGAUD, the MGALD and the MGASD in the DE/cGVHD group without systemic cGVHD was similar to the value of those in the non-DE/non-cGVHD group (Table 6).

## Discussion

To the authors’ knowledge, this is the first comprehensive study to conduct a morphologic assessment of the MG in DE patients with cGVHD after HSCT using in vivo CM. We found that in patients with cGVHD related DE, the MG showed severe abnormalities, as assessed by in vivo CM. The characteristics of MGD in cGVHD are that atrophic MG and excessive fibrosis assessed by CM accumulated the almost entire MG including acini, ductules, main ducts, and orifices. In addition, MGD in cGVHD progressed rapidly shortly after the onset of DE [4].

MGs are specialized sebaceous glands. It has been reported that the bulge of hair follicles located below the opening of the sebaceous duct is destroyed by donor T cells in cGVHD skin lesions [26]. Blisters formation of the mucous membrane due to pseudomembrane or donor lymphocyte and/or donor macrophages attack to the recipient epithelia can occur in ocular cGVHD [27]. Similar pathogenic process might contribute recipient MG epithelia of orifices, ducts, ductule or acini, leading to secondary fibrotic spontaneous occlusion and the adhesion of various structures of meibomian glands.

Previous studies showed that tissue atrophy and excessive fibrosis are prominent histological features of salivary gland and lacrimal gland in cGVHD [28-30]. We have reported T cells and fibroblasts in lacrimal gland cGVHD patients primarily are activated in the periductal area, and contribute to the pathogenic fibrosis [30]. In addition, activated fibroblasts derived from epithelia via local epithelial mesenchymal transition [31] or arise from bone marrow under inflammatory stress [29] might contribute to excessive fibrosis around the MG ducts. Therefore, we proposed that extensive MG fibrosis were exacerbated by inflammation under inflammatory cells and fibroblasts interaction and subsequent activation leading to excessive accumulation of extracellar matrix and pathogenic fibrosis in meibomian gland microenvironment. These factors are considered to be characteristic in cGVHD in comparison with other MGD.

Aging may also exacerbate the periglandular inflammation and fibrosis. Because it is reported that MGD is facilitated by aging [7,22], median age is 50.5 years in our cases may have a potential to develop MGD by aging. However, MGD parameter of age-matched non-GVHD patients was statistically better, the possibility influenced by aging is unlikely. Therefore, we propose that our results obtained this study is associated with cGVHD.

Because we prospectively followed the HSCT recipients since 1996, we could observe the MG at the onset of DE related to cGVHD. Excessive fibrosis was detectable by in vivo CM shortly after the onset of cGVHD-related DE. Previous reports have found that the MGALD (86.3±18.9 μm) and MGASD (34.8±9.2 μm) were both significantly larger in MGD patients than in controls (56.3±10.4 μm, p<0.0001, and 17.4±4.2 μm, p<0.0001, respectively) [11,12]. In age related MGD, the mechanism of morphologic changes in MG was suggested that hyperkeratinization of the ductal epithelium, shedding of keratinized material into the glandular ducts leading to orificial obstructions, and eventual cystic dilatation and atrophy [32].

Recently, morphological changes of MG in Sjogren’s syndrome (SS) have been reported using CM. In this study, MGALD has been reported 53±31μm in primary SS and 70±42 μm in secondary SS. These values are longer than that of cGVHD obtained in this study [33]. In cGVHD, tissue atrophy and excessive fibrosis were prominent histological features, may explain the disaccordance of our results with those of previous reports.

Interestingly, we also found that the mean MGAUD in the DE/cGVHD group with systemic cGVHD was lower than in the DE/cGVHD group without systemic cGVHD, and the mean MGALD and MGASD were shorter. These patients had severe DE, and 5 out of 9 HSCT recipients (55.6%) showed excessive fibrosis of the MG. The MG of patients with DE but without systemic cGVHD resembled those of the non-DE group. A previous report showed that cGVHD patients with conjunctival fibrosis had systemic complications and a poor prognosis following HSCT [14]. Our results suggested that the MG of DE/non-systemic cGVHD resembled those of the non-DE group. In other words, these findings suggested that dry eye related to cGVHD did not affected meibomian gland alterations. Although we analyzed a small number of patients, there is a statistically significant difference for fibrosis grades between the MG of DE/non-systemic cGVHD and those of the non-DE group. There is a possibility that fibrosis rather than dry eye itself may play a central role for the development of the MGD in cGVHD, similar to cGVHD lacrimal gland pathology. Our previous study have insisted that fibrosis is a leading cause of dry eye related to cGVHD and even in the early course of the diseases [34]. In cGVHD, there is a possibility that fibrosis is a primary event and consequently leads to MGD and dry eye separately.

Taken together, we believe that the cause of MGD in cGVHD is multifactorial and multistep. Destruction of the ductal epithelia due to lymphocyte infiltration, slouging of epithelial cells due to lymphocyte attack or pseudomembrane formation, and subsequent excessive fibrosis around the orifice, ducts, ductules, and acini of the MG, may explain for the development of MGD after HSCT.

Although further studies are necessary, our study suggests that CM findings on the MG in HSCT recipients may be useful for evaluating their likely progression to MGD. In this study, we conducted CM for the lower lid. It is difficult for us to evaluate the upper eyelid margin, whereas the lower one is easily positioned and examined. A confocal examination require a prolonged contact for several minutes between instrument and examined inverted upper eye lid tarsal conjunctiva, which can be uncomfortable for the patient during examination as reported previously [33]. Moreover the instrument has a space fix orientation, so it requires the polymethacrylate sterile cap (Tomo-cap) face, leads to more difficult to evaluate the upper lid meibomian glands. It is necessary for us to develop the methods as for examining upper tarsal conjunctiva more comfortable for further evaluating the entire MG morphology.

We used topical immunosuppressants when the patients were tapering off their systemic immunosuppressive therapy for cGVHD related dry eye [35]. Furthermore, our group reported that topical tranilast for the treatment of early fibrosis may effectively retard cGVHD related DE progression when given in the early stages [36]. Therefore, early use of topical immunosuppressant and anti-fibrotic intervention might be useful strategy for preventing or retarding the MGD associated fibrosis in cGVHD.

In summary, we conducted a morphometric assessment of the MG in DE patients with cGVHD using in vivo laser CM. CM clearly demonstrated morphological changes including inflammatory cell infiltration and excessive fibrosis, even in early stage, in the MG of DE patients with cGVHD. Our results also suggested that MGD may allow us to diagnose severe DE and cGVHD early in the course of the disease. Early detection of MGD could be important, because it could signal a progression to severe DE, and extensive cGVHD, which can lead to blindness and become life-threatening.
